# Primary acute lymphoblastic leukemia cells are susceptible to microtubule depolymerization in G1 and M phases through distinct cell death pathways

**DOI:** 10.1016/j.jbc.2022.101939

**Published:** 2022-04-15

**Authors:** Magdalena Delgado, Randall R. Rainwater, Billie Heflin, Alicja Urbaniak, Kaitlynn Butler, Mari Davidson, Reine M. Protacio, Giulia Baldini, Andrea Edwards, Megan R. Reed, Kevin D. Raney, Timothy C. Chambers

**Affiliations:** Department of Biochemistry and Molecular Biology, University of Arkansas for Medical Sciences, Little Rock, Arkansas, USA

**Keywords:** microtubule depolymerization, acute lymphoblastic leukemia, cell death, apoptosis, Bcl-2 proteins, parylation, apoptosis-inducing factor, EndoG, autophagy, AIF, apoptosis-inducing factor, AP, autophagosomes, AL, autolysosomes, ALL, acute lymphoblastic leukemia, Cdk, Cyclin-dependent kinase, EndoG, endonuclease G, FCCP, carbonyl cyanide 4-(trifluoromethoxy)phenylhydrazone, GAPDH, glyceraldehyde 3-phosphate dehydrogenase, MLKL, mixed lineage kinase domain like pseudokinase, MTA, microtubule targeting agent, PARP, poly (ADP)-ribose polymerase, VCR, vincristine

## Abstract

Microtubule targeting agents (MTAs) are widely used cancer chemotherapeutics which conventionally exert their effects during mitosis, leading to mitotic or postmitotic death. However, accumulating evidence suggests that MTAs can also generate death signals during interphase, which may represent a key mechanism in the clinical setting. We reported previously that vincristine and other microtubule destabilizers induce death not only in M phase but also in G1 phase in primary acute lymphoblastic leukemia cells. Here, we sought to investigate and compare the pathways responsible for phase-specific cell death. Primary acute lymphoblastic leukemia cells were subjected to centrifugal elutriation, and cell populations enriched in G1 phase (97%) or G2/M phases (80%) were obtained and treated with vincristine. We found death of M phase cells was associated with established features of mitochondrial-mediated apoptosis, including Bax activation, loss of mitochondrial transmembrane potential, caspase-3 activation, and nucleosomal DNA fragmentation. In contrast, death of G1 phase cells was not associated with pronounced Bax or caspase-3 activation but was associated with loss of mitochondrial transmembrane potential, parylation, nuclear translocation of apoptosis-inducing factor and endonuclease G, and supra-nucleosomal DNA fragmentation, which was enhanced by inhibition of autophagy. The results indicate that microtubule depolymerization induces distinct cell death pathways depending on during which phase of the cell cycle microtubule perturbation occurs. The observation that a specific type of drug can enter a single cell type and induce two different modes of death is novel and intriguing. These findings provide a basis for advancing knowledge of clinical mechanisms of MTAs.

Microtubule targeting agents (MTAs) including the taxanes and vinca alkaloids are widely used in cancer chemotherapy (1, 2). These drugs bind to microtubules or tubulin and induce mitotic arrest and intrinsic apoptosis (3, 4, 5). Intrinsic (or mitochondrial) apoptosis is regulated by the Bcl-2 protein family which exhibit either prosurvival or proapoptotic properties (6, 7, 8). Prosurvival members include Bcl-2, Bcl-xL, Mcl-1, Bcl-w, and Bfl-1/A1, and proapoptotic members include the multidomain effectors Bax, Bak, and Bok, and the BH3-only subfamily comprised of activators (Bim and Bid) and sensitizers (Bad, Noxa, Bik, Bmf, Hrk, and others). Bcl-2 proteins play an important role in mitotic-arrest–induced apoptosis (9). Our studies using HeLa and several other cell lines, for example, have shown that MTAs cause Bax and Bak activation, leading to cytochrome c release from the mitochondria and caspase-3 activation, and that cells lacking Bax and Bak, or overexpressing Bcl-2 or Bcl-xL, are highly resistant to MTAs (10, 11, 12, 13). MTAs also induce phosphorylation of prosurvival members Bcl-2, Bcl-xL, and Mcl-1 during mitotic arrest. Phosphorylation of Bcl-2 and Bcl-xL disables prosurvival function by reducing affinity for select proapoptotic Bcl-2–binding partners (12, 14), and phosphorylation of Mcl-1 is associated with its degradation (11, 15), a process that regulates the kinetics of mitotic death (16, 17).

Whether MTAs strictly target mitotic cells in clinical settings has been under debate (18, 19, 20, 21, 22, 23). The lack of clinical efficacy of drugs that target mitotic kinases and mitotic motor proteins, together with the extended doubling time and low mitotic indices of tumors that are susceptible to MTAs, has led to the hypothesis that MTAs may primarily target interphase microtubules rather than mitotic spindles (18, 19, 24). However, since cultured cancer cell lines are typically only susceptible to MTAs in mitosis, relevant laboratory models are lacking. We therefore screened a large selection of cultured cancer cells and discovered that primary adult acute lymphoblastic leukemia (ALL) cells are susceptible to clinically relevant concentrations of MTAs not only in M phase but also in G1 phase (25). These studies were facilitated by the use of centrifugal elutriation which enabled the isolation of cells highly enriched in either G1 or G2/M phases (26). Subsequent investigation showed that death directly in G1 phase required active cell cycle advance and did not occur in G1-arrested cells (27). Furthermore, G1 phase death of primary ALL cells was found to occur only in response to microtubule destabilizing drugs under conditions of complete microtubule depolymerization and was not observed after treatment with microtubule stabilizing drugs where polymerized microtubule structures were retained (28). In addition, of cells in interphase, only those in G1 phase were susceptible to microtubule depolymerization; cells in S or G2 phases were not, instead progressing to M phase where they arrested and died (25). From these observations, we concluded that functional microtubules are essential for successful G1 phase advance of primary ALL cells, possibly due to their role in trafficking key proteins needed for S phase to the nucleus, but are dispensable for G1-arrested cells that are not preparing for S phase (27).

The availability of primary ALL cells enriched in either G1 or G2/M phases provided an opportunity to investigate and compare the death pathway(s) activated in response to microtubule destabilization. Numerous mechanisms of cell death have been recognized including apoptosis, necrosis, necroptosis, ferroptosis, parthanatos, autophagy-dependent, and many others (29). Here we report the results of such a study which shows that while certain features are common to both, distinct pathways are responsible for death of G1 *versus* M phase primary ALL cells in response to vincristine (VCR). To the best of our knowledge, these results are the first to demonstrate that a specific drug can enter a single cell type and induce two different forms of cell death. The findings provide a basis for advancing our understanding of clinical mechanisms of MTAs.

## Results

### Cell cycle analysis of elutriated ALL cells

**Figure 1:**
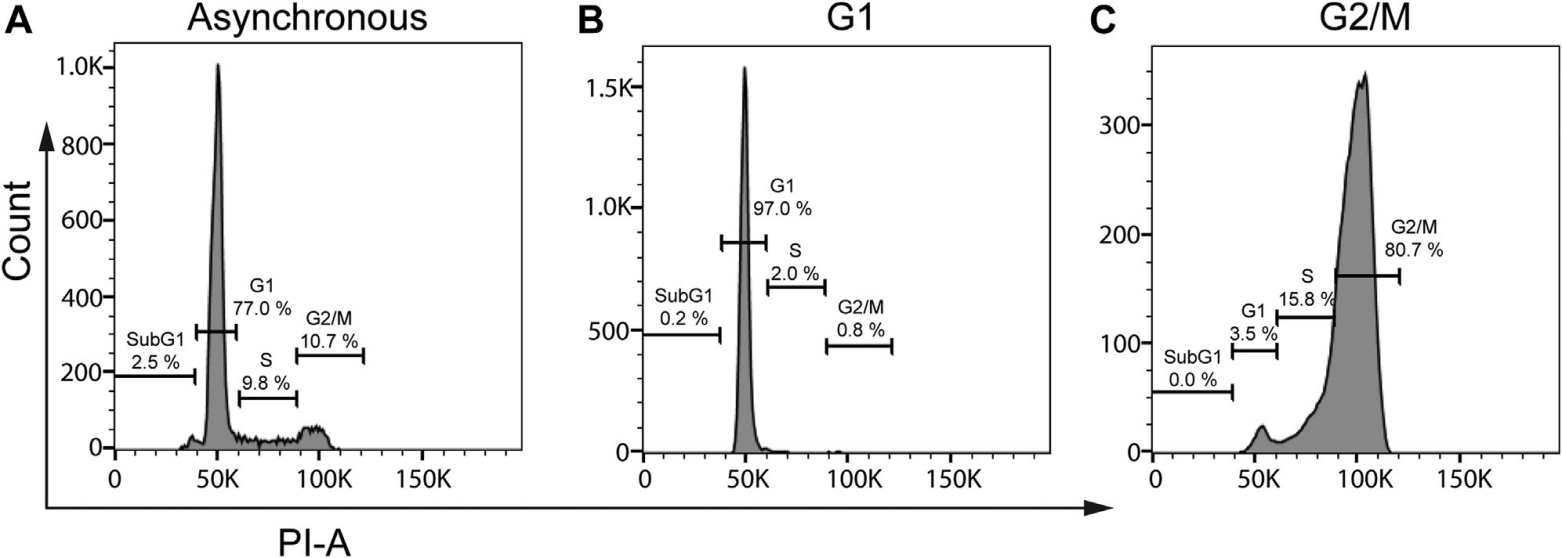
**Analysis of DNA content of phase-purified ALL cells.** Asynchronous ALL-5 cells were subjected to centrifugal elutriation and pools corresponding to cells in different cell cycle phases obtained, as described in Experimental procedures. Cells were stained with propidium iodide and subjected to flow cytometry to determine DNA content as an indicator of cell cycle phase purity. *A*, Asynchronous cells; *B*, G1 phase (2N DNA); *C*, G2/M phases (4N DNA). The results shown are representative of numerous (>40) experiments.

Primary ALL cells were subjected to centrifugal elutriation as described in Experimental procedures and fractions corresponding to cells in G1 phase or G2/M phases were prepared and stained with propidium iodide to determine DNA content and verify purity. Representative data are shown in Figure 1, together with that of the initial asynchronous population. The majority of cells in asynchronous culture were in G1 phase containing 2N DNA, with much smaller proportions in S and G2/M phases with >2N-4N DNA content (Fig. 1*A*). Asynchronous cultures also routinely contained a small proportion (∼2.5%) of dead or dying cells indicated by sub-G1 DNA content. The G1 phase pool was highly enriched in cells with 2N DNA content (97%), with extremely low contamination of cells in other cell cycle phases (Fig. 1*B*). The G2/M pool was also greatly enriched in cells with 4N DNA content (>80%), with relatively low levels of cells in other phases (Fig. 1*C*). Importantly, primary ALL cells in S or G2 phases when treated with VCR or other microtubule destabilizers transit to M phase where they arrest and die (25, 28). Thus, over 96% of cells in the designated G2/M fraction are destined to undergo mitotic death upon drug treatment. Conversely, the majority of cells in the G1 phase fraction, when treated with VCR or other microtubule destabilizers at concentrations of ≥30 nM, die directly in G1 (25, 28). Thus, the two fractions generated by centrifugal elutriation are ideally suited to the purpose of this study to compare the respective phase-specific death pathways.

### Differential role of Bcl-2 proteins

**Figure 2:**
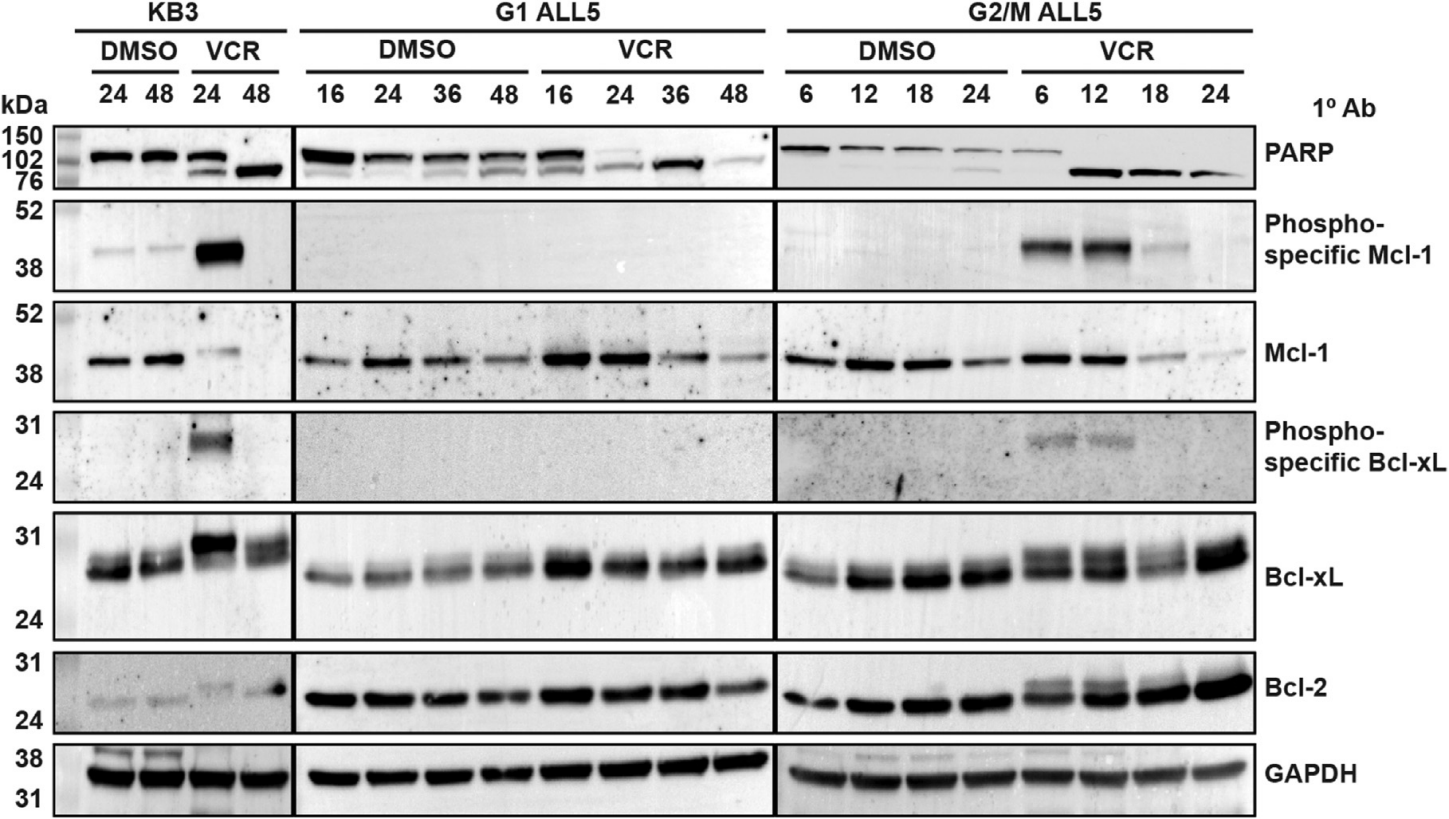
**Vincristine-induced death of G2/M but not G1 phase ALL cells is associated with phosphorylation of pro-survival Bcl-2 proteins.** G1 and G2/M phase ALL cells were isolated by centrifugal elutriation and treated with vehicle (0.1% DMSO) or 100 nM vincristine (VCR) for the times (h) indicated. Asynchronous KB-3 cells were also treated as indicated. Whole cell extracts were prepared and subjected to immunoblot analyses for PARP, total Mcl-1, phospho-Mcl-1, total Bcl-xL, phospho-Bcl-xL, total Bcl-2, or GAPDH as a loading control. Molecular mass standards are indicated on the left; molecular masses of the individual proteins are given in Experimental procedures. Images are representative of three independent experiments. ALL, acute lymphoblastic leukemia; PARP, poly (ADP)-ribose polymerase.

Previous studies by us and others have shown that Bcl-2 proteins play a key role in mitotic death (10, 11, 12, 13, 14, 15, 16, 17), consistent with observations that cell death in response to mitotic arrest occurs largely *via* intrinsic apoptosis (3, 4, 5). We therefore first examined the expression, phosphorylation, and activation status of members of the Bcl-2 protein family. G1 or G2/M phase ALL cells were prepared and treated with vehicle (0.1% DMSO) or 100 nM VCR. Previously, it was shown that death of cells in G2/M phases in response to VCR, based on assessment of poly (ADP)-ribose polymerase (PARP) cleavage, occurred more rapidly than that of cells in G1 phase (25). Therefore, drug exposure times differed, with periods up to 48 h for G1 cells and up to 24 h for G2/M cells. KB-3 cells, a HeLa subline, were used as a control for mitotic death because we have shown previously that in response to MTAs, they exhibit a well-defined mitotic arrest followed by intrinsic apoptosis (30, 31). As shown in Figure 2, left panel, VCR induced time-dependent cleavage of PARP in KB-3 cells, while PARP remained intact in vehicle-treated cells. VCR also induced phosphorylation of both Mcl-1 and Bcl-xL at 24 h, indicated by mobility shifts of these proteins on immunoblots employing phosphorylation-independent antibodies, as well as by detection using phospho-specific antibodies (Fig. 2). In the case of Bcl-xL, phosphorylation was reversed by 48 h, whereas in the case of Mcl-1, phosphorylation resulted in loss of Mcl-1 expression, determined previously to be due to proteosome-mediated degradation (32). While Mcl-1 levels were decreased after VCR treatment as evaluated by use of a phosphorylation-independent antibody, strong immunoreactivity with the phospho-specific Mcl-1 antibody was retained. This is due to the high level of Mcl-1 phosphorylation, at up to nine sites, as we reported previously (33). Bcl-2 is only weakly expressed in KB-3 cells but nonetheless VCR induced a transient mobility shift, previously determined to be due to phosphorylation (31). Overall similar changes were noted when G2/M phase ALL cells were treated with VCR (Fig. 2, right panel). Thus, PARP underwent cleavage, and phosphorylation of Mcl-1, Bcl-xL, and Bcl-2 was observed, together with loss of Mcl-1 expression. In contrast, when ALL cells in G1 phase were treated with VCR (Fig. 2, center panel), PARP cleavage was not accompanied by phosphorylation of prosurvival Bcl-2 proteins, and while Mcl-1 expression was reduced, it occurred in a phosphorylation-independent manner.

**Figure 3:**
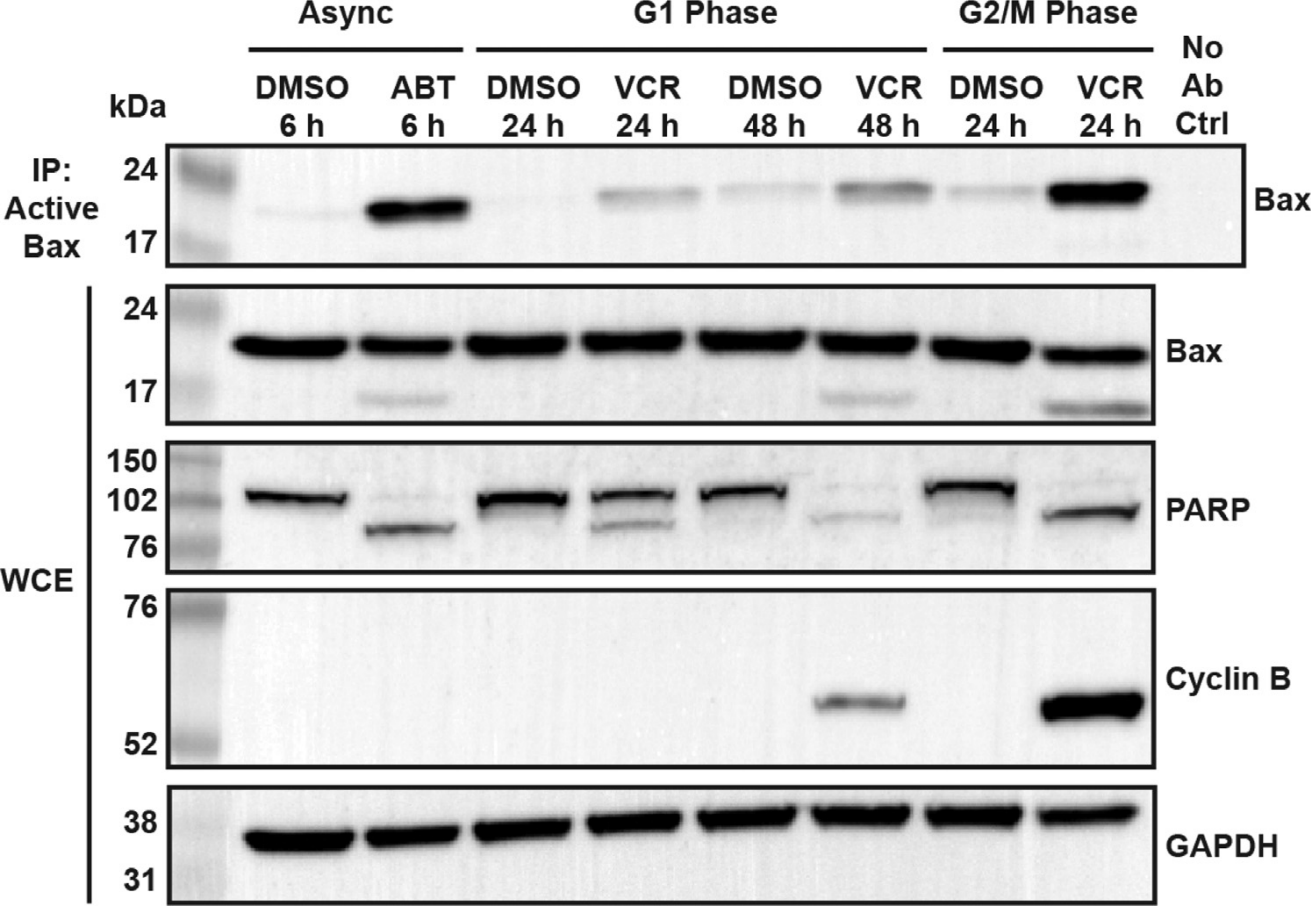
**Vincristine treatment strongly activates Bax in G2/M but not G1 phase ALL cells.** Asynchronous ALL-5 cells were treated with 100 nM ABT-263 for 6 h as a positive control for Bax activation. G1 and G2/M phase ALL-5 cells were isolated using centrifugal elutriation and treated with vehicle (0.1% DMSO) or 100 nM VCR for the times indicated. Samples were lysed under nondenaturing conditions, and lysates were subjected to immunoprecipitation for active Bax using 6A7 antibody, which recognizes the active conformation of Bax. Immunoprecipitated samples (IP) and whole cell extracts (WCEs) were analyzed by immunoblotting for Bax, PARP, cyclin B, or GAPDH as a loading control. Mock immunoprecipitation (No Ab Ctrl, *upper right lane*) was conducted as described in Experimental procedures. ALL, acute lymphoblastic leukemia; VCR, vincristine.

Next, Bax activation was examined, as described in Experimental procedures. ALL cells in G1 or G2/M phases were prepared and treated with vehicle or 100 nM VCR. Extracts were made under native conditions and subjected to immunoprecipitation with 6A7 active Bax antibody, followed by immunoblotting for Bax. Asynchronous ALL cells treated with the Bcl-2/Bcl-xL inhibitor ABT-263, which induces rapid intrinsic apoptosis by disrupting Bcl-2/Bcl-xL suppression of prodeath Bcl-2 proteins (34), was used as a positive control. As shown in Figure 3, ABT-263 induced strong Bax activation in asynchronous cells as expected, and VCR also induced strong Bax activation in G2/M phase cells, and this occurred in concert with PARP cleavage. However, only weak Bax activation was associated with PARP cleavage in G1 phase cells treated with VCR for 24 or 48 h. Total Bax levels in whole cell extracts remained relatively unchanged under all conditions (Fig. 3). Our earlier studies showed that while the majority of G1 phase ALL cells treated with VCR died directly from G1 phase, a small proportion that varied from 5 to 15% escaped direct death and advanced in the cell cycle and died *via* mitotic death (25). Since cyclin B expression is a characteristic marker of mitosis (35), the same extracts were probed for cyclin B. Strong cyclin B expression was observed in G2/M phase cells treated with VCR as anticipated, and weak cyclin B expression was observed in VCR-treated G1 cells (Fig. 3). Band intensities were quantified by Image J analysis, and the level of Bax activation after VCR treatment in G1 phase cells was determined to be about 20% that of G2/M cells. Similarly, the level of cyclin B expression after VCR treatment of G1 cells was found to be about 20% that of G2/M cells. These results strongly suggest that the low level of Bax activation observed in drug-treated G1 phase cells derives from the small proportion of cells that escape direct death and advance in the cell cycle and undergo mitotic death and strengthen the notion that G1 phase death is Bax-independent.

### Differential activation of caspase-3

**Figure 4:**
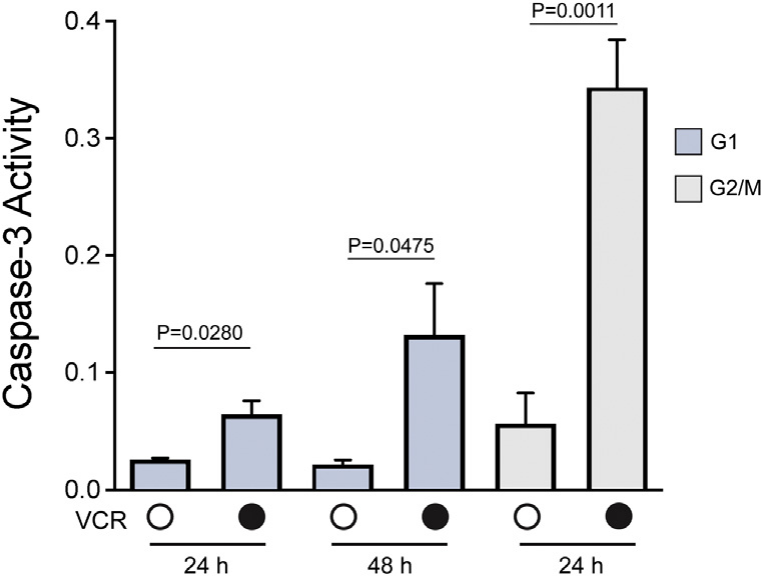
**Vincristine activates caspase-3 more strongly in G2/M than G1 phase cells.** G1 phase and G2/M phase ALL-5 cells were isolated using centrifugal elutriation and treated with vehicle (0. 1% DMSO) or 100 nM VCR for the times indicated. Protein extracts were prepared and caspase-3 activity determined as described in Experimental procedures. Data shown are mean ± S.D. (n ≥ 3) with *p* values indicated. ALL, acute lymphoblastic leukemia; VCR, vincristine.

Intrinsic apoptosis is associated with activation of initiator and executioner caspases including caspase-3 (36). Cell extracts prepared after VCR treatment of G1 or G2/M phase ALL-5 cells were subjected to caspase-3 assay, as described in Experimental procedures. Asynchronous ALL-5 cells treated with the Bcl-2 inhibitor ABT-263 were used as a positive control. ABT-263 treatment strongly and rapidly activated caspase-3, and this was completely inhibited by cotreatment with the pan caspase inhibitor Z-VAD-FMK (Fig. S1). VCR strongly activated caspase-3 in G2/M phase cells after 24 h treatment but only weakly activated caspase-3 in G1 phase cells at 24 h (Fig. 4). To exclude the possibility that caspase-3 activation was delayed in G1 phase cells, assays were also conducted at 48 h of VCR treatment, and levels considerably lower than G2/M phase cells were still observed (Fig. 4). A highly comparable profile of results were obtained when an independent culture of primary ALL cells, ALL-2, which were derived from a different patient (37), were used (Fig. S2).

### Differential effects of caspase inhibition on DNA fragmentation

**Figure 5:**
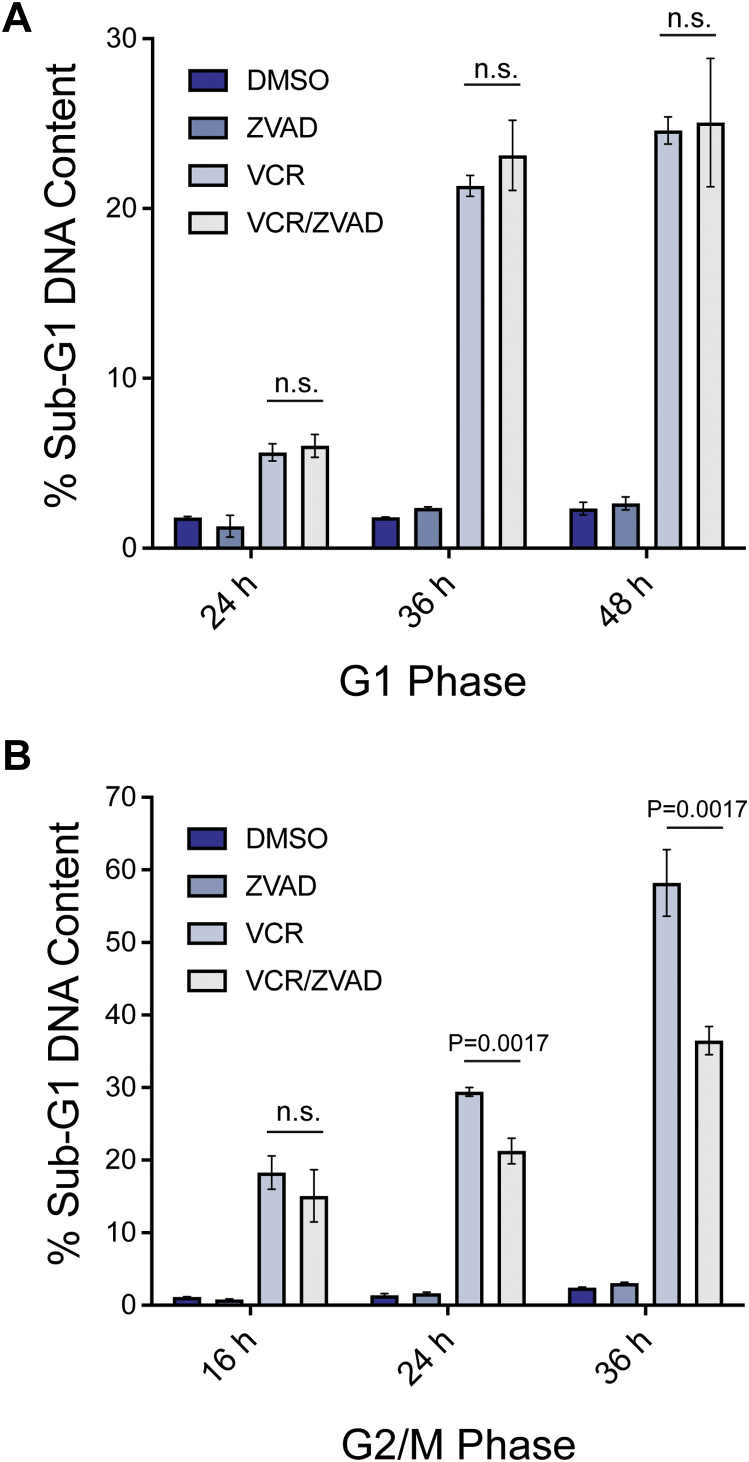
**Differential effects of caspase inhibition on DNA fragmentation in G1 *versus* G2/M phase cells after vincristine treatment**. *A*, G1 or *B*, G2/M phase ALL-5 cells, isolated *via* centrifugal elutriation, were treated with 100 nM VCR, 100 μM Z-VAD-FMK, or vehicle (0.1% DMSO), alone or in combination, as indicated. Cells were harvested and stained with propidium iodide and analyzed for DNA content as described in Experimental procedures. Data shown represent percent of cells with sub-G1 DNA content (mean ± SD, n = 3). *p* values are indicated; n.s. = not significantly different. ALL, acute lymphoblastic leukemia; VCR, vincristine.

DNA fragmentation during intrinsic apoptosis is in part caspase-dependent, through a mechanism whereby caspases degrade the inhibitor of the responsible nuclease thus activating it (38, 39). Since caspase-3 was differentially activated by VCR in G1 *versus* G2/M phase ALL cells, we sought to determine whether differences existed with respect to caspase dependence of DNA fragmentation. Asynchronous ALL-5 cells treated with the Bcl-2 inhibitor ABT-263 were used as a positive control, and DNA fragmentation was assessed by sub-G1 DNA content after propidium iodide staining and flow cytometry. As shown in Fig. S3, the caspase inhibitor Z-VAD-FMK completely inhibited DNA fragmentation induced by ABT-263. In contrast, caspase inhibition had no effect on DNA fragmentation induced by VCR in G1 phase cells (Fig. 5*A*), whereas partial and highly statistically significant inhibition was observed in VCR-treated G2/M cells (Fig. 5*B*). These results suggest that caspase activation plays a significant role in DNA fragmentation during mitotic death of primary ALL cells but plays no role in DNA fragmentation of G1 phase cells in response to microtubule depolymerization.

### G1 phase and mitotic death pathways differ with respect to DNA cleavage products

**Figure 6:**
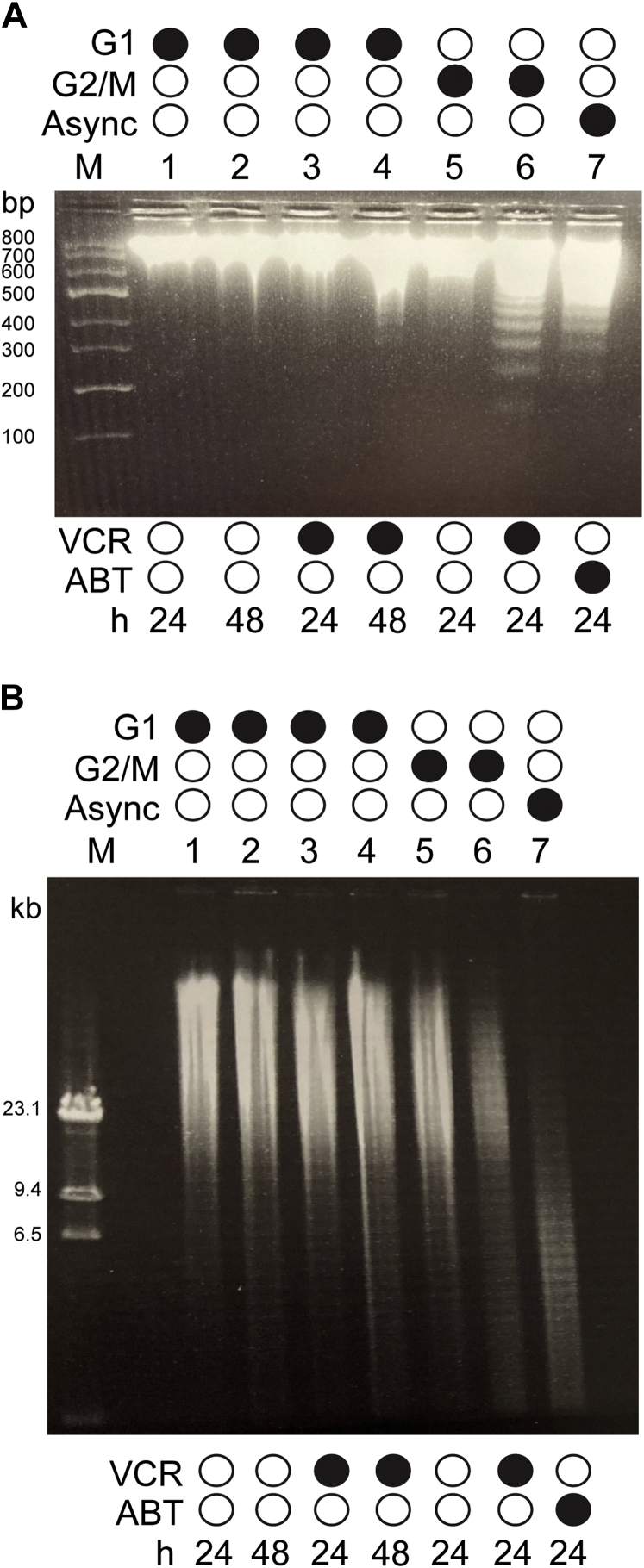
**Analysis of DNA fragmentation by gel electrophoresis**. *A*, analysis of small fragments. DNA was prepared as described in Experimental procedures and samples (8 μg) separated by 2% agarose gel electrophoresis. Lanes are as indicated and as follows: M, DNA standards, with molecular masses indicated at left; 1, G1 phase cells, vehicle (0.1% DMSO), 24 h; 2, G1 phase cells, vehicle, 48 h; 3, G1 phase cells, 100 nM VCR, 24 h; 4, G1 phase cells, 100 nM VCR, 48 h; 5, G2/M phase cells, vehicle, 24 h; 6, G2/M phase cells, 100 nM VCR, 24 h; 7, asynchronous cells, 100 nM ABT-263, 24 h. *B*, analysis of large fragments. DNA was prepared as described in Experimental procedures and samples (0.2 μg) separated by pulsed field gel electrophoresis. Lanes as in panel A. Gels were stained with ethidium bromide. ALL, acute lymphoblastic leukemia; VCR, vincristine.

While propidium iodide staining and flow cytometry provide a means to quantitate DNA fragmentation, the technique does not provide information on the size of the DNA fragments generated. Intrinsic apoptosis results in the formation of small nucleosomal DNA fragments which characteristically produce a 200 bp ladder when analyzed by gel electrophoresis (40). In contrast, other forms of cell death, for example parthanatos, result in activation of nucleases which produce much larger DNA fragments (41, 42). We therefore examined DNA fragment size by gel electrophoresis. ALL cells in G1 or G2/M phases were isolated, treated with VCR, and DNA prepared, as described in Experimental procedures. Small DNA fragments were first examined using 2% agarose gels, and they were not observed after vehicle treatment of G1 phase cells as expected (Fig. 6*A*, lanes 1 and 2) nor were they detected after VCR treatment of G1 phase cells for 24 h (lane 3) or 48 h (lane 4). However, low molecular weight DNA fragments forming a characteristic ladder were readily observed after VCR treatment of G2/M phase cells (Fig. 6*A*, lane 6). Similarly, ABT-263 treatment of asynchronous ALL cells produced a pattern of DNA laddering characteristic of intrinsic apoptosis (Fig. 6*A*, lane 7). To determine if VCR treatment of G1 phase ALL cells produced large DNA fragments, DNA was prepared and analyzed by pulsed field gel electrophoresis (Fig. 6*B*). However, we were unable to resolve discrete high molecular weight fragments, and instead DNA from both DMSO-treated (Fig. 6*B*, lanes 1 and 2) and VCR-treated (lanes 3 and 4) G1 phase ALL cells appeared as an indistinct smear of DNA staining. In contrast, after VCR treatment of G2/M cells (Fig. 6*B*, lane 6) or ABT-263 treatment of asynchronous cells (lane 7), the intensity of staining of high molecular weight DNA species was clearly diminished compared to vehicle treatment (lane 5), consistent with the generation of smaller fragments. Thus, VCR-induced death of G1 phase ALL cells is associated with a persistence of high molecular weight DNA and an absence of low molecular weight DNA fragments, whereas the pattern of DNA fragmentation in VCR-treated G2/M cells is consistent with apoptotic death.

### Necroptosis does not play a role

We next considered whether G1 phase death in response to VCR occurred *via* necroptosis. Necroptosis involves sequential activation of RIPK3 and mixed lineage kinase domain like pseudokinase (MLKL) which culminates in plasma membrane permeabilization and can lead to nonspecific cleavage of DNA (43). A defining feature of necroptosis is phosphorylation of MLKL which can be readily observed by immunoblotting with phospho-specific antibodies. G1 phase ALL cells were prepared by centrifugal elutriation, treated with VCR for periods up to 48 h, and cell extracts subjected to immunoblotting with a phospho-specific MLKL antibody. HT-29 colon carcinoma cells treated with a combination of TNF-α, cycloheximide, and Z-VAD-FMK were used a positive control (44). As shown in Fig. S4, phosphorylation of MLKL was observed in treated HT-29 cells but was not observed in G1 phase ALL cells after VCR treatment, suggesting that necroptosis does not play a role.

### Both death pathways involve loss of mitochondrial transmembrane potential

**Figure 7:**
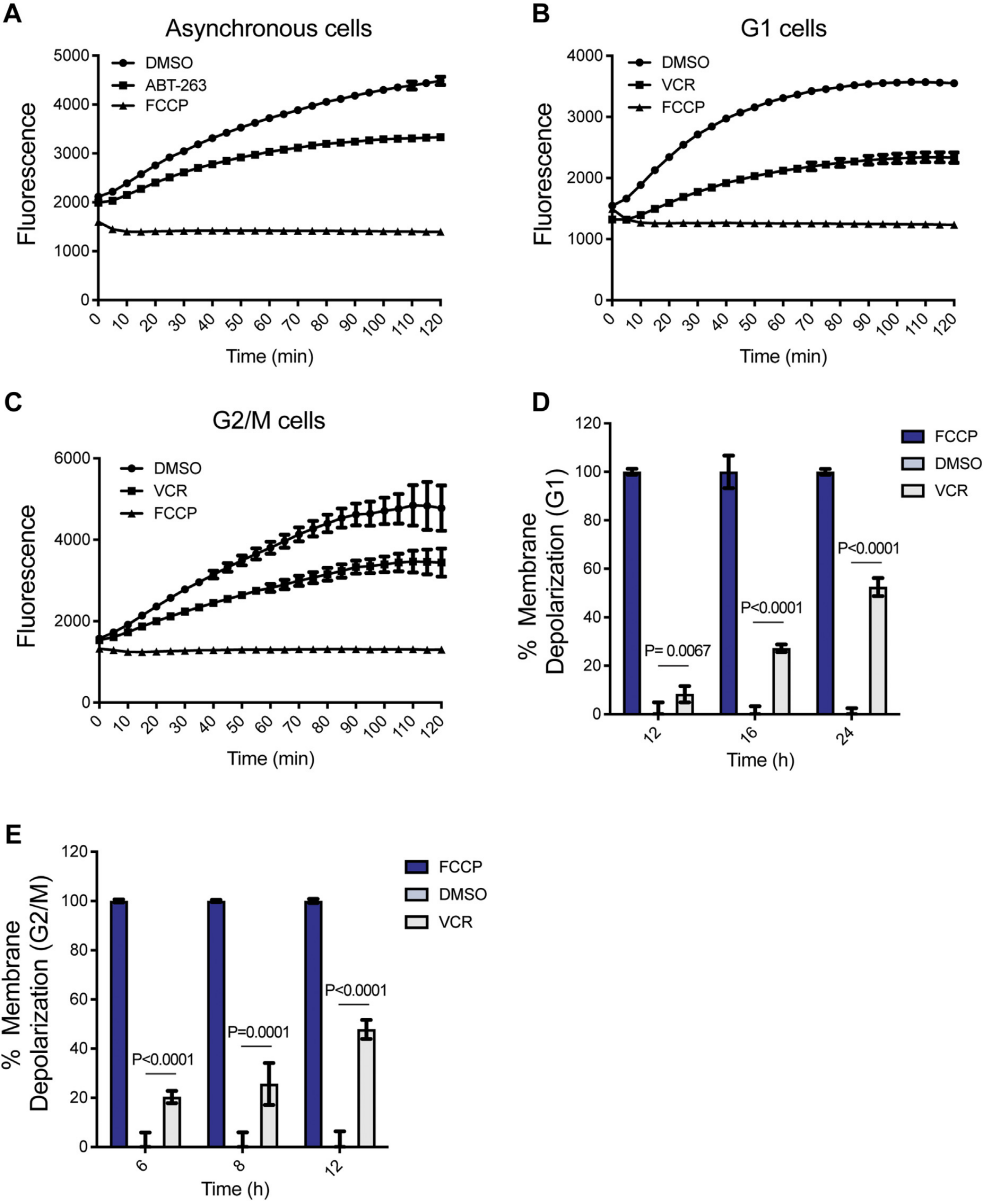
**Vincristine induces loss of mitochondrial transmembrane potential in both G1 and G2/M phase ALL cells**. Changes in mitochondrial membrane potential were determined by measuring JC-1 fluorescence, as described in Experimental procedures. Panels *A*–*C* show fluorescent traces, with respect to time of incubation with JC-1, derived from the following treatment conditions. In all cases, 0.1% DMSO was used as the vehicle control and 10 μM FCCP as a positive control for decay of fluorescence. *A*, asynchronous ALL-5 cells treated with ABT-263 for 60 min; *B*, G1 phase cells treated with 100 nM VCR for 24 h; *C*, G2/M phase cells treated with 100 nM VCR for 12 h. Panels *D* and *E* show results (mean ± S.D., n = 3, with *p* values indicated) normalized to that of FCCP (100% loss of mitochondrial membrane potential) derived from tracings at 120 min incubation with JC-1 for either: *D*, G1 phase cells, or *E*, G2/M phase cells, treated with 100 nM VCR or 0.1% DMSO for the times indicated. ALL, acute lymphoblastic leukemia; FCCP, carbonyl cyanide 4-(trifluoromethoxy)phenylhydrazone; VCR, vincristine.

Mitochondria play a key role in both caspase-dependent and caspase-independent death pathways (45, 46). During intrinsic apoptosis, activation of effector Bcl-2 proteins such as Bax and Bak creates pores in the outer mitochondrial membrane and the release of cytochrome c, and other forms of cell death involve release from the mitochondria of prodeath factors such as apoptosis-inducing factor (AIF) and endonuclease G (EndoG) (41, 42, 45, 46). These events are accompanied by mitochondrial outer membrane permeabilization and loss of mitochondrial transmembrane potential, which can be measured by changes in fluorescence of the cationic dye JC-1 (47). In healthy cells, JC-1 accumulates in the mitochondria and forms red fluorescent aggregates, whereas in unhealthy or dying cells, JC-1 fails to form aggregates and exhibits green fluorescence. ALL cells in G1 or G2/M phases were prepared, treated with VCR, incubated with JC-1, and fluorescence measured as described in Experimental procedures. Asynchronous ALL cells treated with ABT-263 provided a positive control for mitochondrial outer membrane permeabilization, and treatment of cells with carbonyl cyanide 4-(trifluoromethoxy)phenylhydrazone (FCCP), a protonophore which completely depolarizes mitochondrial membranes, was used to normalize data. The data are presented in Figure 7, with representative graphs in panels A-C and data quantitation in panels D and E. As shown in Figure 7*A*, in vehicle-treated asynchronous cells, fluorescence in the red spectrum at 590 nm increased with time, as JC-1 accumulated as aggregates in healthy mitochondria. In cells treated with ABT-263, there was a marked decrease in fluorescence compared to vehicle-treated cells. In the presence of FCCP, the curve remained flat, and no increase was observed, as expected. In G1 phase, cells treated for 24 h with VCR, fluorescence was markedly diminished compared to cells treated with vehicle (Fig. 7*B*). A more extensive time course of VCR treatment in G1 phase cells was conducted and a time-dependent increase in mitochondrial membrane depolarization was observed which reached about 50% of that observed in the presence of FCCP, which was set at 100% (Fig. 7*D*). Similarly, in G2/M phase cells treated for 12 h with VCR, fluorescence was markedly diminished compared to cells pretreated with vehicle (Fig. 7*C*) in a manner dependent on time of VCR treatment (Fig. 7*E*). These results indicate that VCR causes loss of mitochondrial transmembrane potential in both G1 and G2/M phase ALL cells, implicating mitochondrial membrane depolarization in both forms of death.

### G1 phase death is associated with nuclear translocation of AIF and EndoG

**Figure 8:**
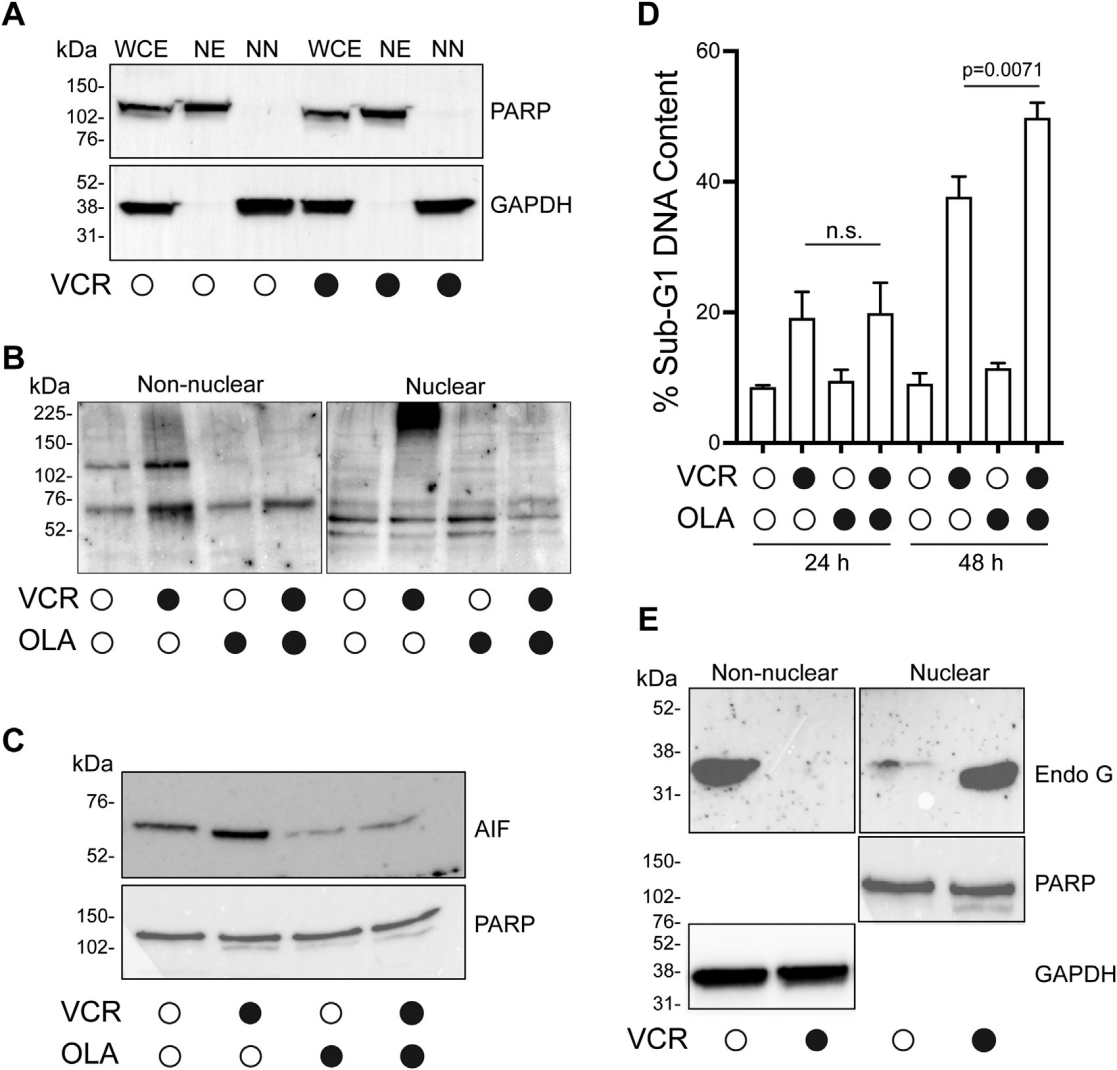
**Death of G1 phase ALL cells in response to vincristine is associated with parylation and nuclear translocation of AIF and EndoG.***A*, non-nuclear (NN) and nuclear (NE) extracts were prepared from control and VCR (100 nM, 16 h)-treated ALL cells in G1 phase as described in Experimental procedures, and 20 μg protein/lane analyzed by immunoblotting for the indicated proteins. WCE, whole cell extract. *B*, G1 phase ALL cells were treated with vehicle (0.1% DMSO), 100 nM VCR, or 3 μM olaparib, alone and in combination, for 16 h, and non-nuclear and nuclear extracts prepared. Extracts (40 μg protein) were subjected to immunoblotting using an antibody that recognizes PAR polymers. *C*, nuclear extracts from A were subjected to immunoblotting for AIF and PARP as indicated. *D*, effect of olaparib on VCR-induced DNA fragmentation. ALL-5 cells were treated with vehicle, 100 nM VCR, or 3 μM olaparib, alone and in combination, for 24 h or 48 h, as indicated, and sub-G1 DNA content determined by propidium iodide staining and flow cytometry, as described in Experimental procedures. Results shown are mean ± S.D., n = 3; n.s. = not significantly different. *E*, non-nuclear and nuclear extracts from A were subjected to immunoblotting for EndoG, PARP, and GAPDH, as indicated. For panels *A*–*C*, and *E*, molecular mass standards are shown at the left. AIF, apoptosis-inducing factor; ALL, acute lymphoblastic leukemia; EndoG, endonuclease G; PARP, poly (ADP)-ribose polymerase; VCR, vincristine.

The results described above indicated that death of G1 phase ALL cells in response to microtubule destabilization occurs in a manner that lacks many of the characteristics of intrinsic apoptosis but was associated with loss of mitochondrial transmembrane potential as well as supranucleosomal fragmentation of DNA. These features suggested parthanatos as a possible mechanism of cell death. Parthanatos involves activation of PARP-1, the generation of poly(ADP)-ribose (PAR) polymers, parylation of target proteins including AIF, release of AIF from the mitochondria and its translocation to the nucleus, and AIF-mediated nuclear DNA fragmentation (41). Because AIF translocates from the mitochondria to the nucleus *via* the cytosol, we prepared nuclear and non-nuclear fractions, as described in Experimental procedures, the latter containing cytosol and smaller organelles, to examine possible effects of VCR on AIF subcellular location. The integrity of the subcellular fractions was first verified in vehicle and VCR-treated G1 phase ALL cells. The cytosolic protein glyceraldehyde 3-phosphate dehydrogenase (GAPDH) partitioned with the non-nuclear fraction and was absent from the nuclear fraction, and the converse was true for the nuclear protein PARP (Fig. 8*A*). Next, G1 phase ALL cells were treated with vehicle or VCR, subcellular extracts were made, and subjected to immunoblotting using an antibody that recognizes PAR polymers. In addition, vehicle or VCR-treated cells were co-treated with olaparib, a PARP-1 inhibitor (48). As shown in Figure 8*B*, VCR induced parylation of certain proteins in both fractions. Several of these appeared to be nonspecific since they were not eliminated in cells co-treated with olaparib. However, an immunoreactive band of about 120 kDa in the non-nuclear fraction, and a very prominent high molecular weight band in the nuclear fraction, were completely eliminated in the presence of olaparib. These results indicate that VCR induces parylation of select proteins in G1 phase ALL cells. Next, nuclear fractions were subjected to immunoblotting for AIF (Fig. 8*C*). VCR induced a marked increase in nuclear AIF levels which was blocked by cotreatment with olaparib, with equal loading confirmed by immunoblotting for PARP. To determine whether inhibition of parylation and inhibition of nuclear translocation of AIF by olaparib influenced cell death by VCR, G1 phase ALL cells were treated with VCR in the presence or absence of olaparib, and DNA fragmentation quantitated by propidium iodide staining and flow cytometry. As shown in Figure 8*D*, olaparib did not inhibit VCR-mediated DNA fragmentation, and at 48 h of treatment, olaparib significantly increased VCR-induced sub-G1 DNA content. Importantly, death mechanisms that induce AIF-mediated DNA fragmentation are also often associated with translocation of the endonuclease EndoG from the mitochondria to the nucleus (42, 45, 46). We therefore examined EndoG and found that it was present in the non-nuclear fraction and absent in the nuclear fraction in vehicle-treated cells but quantitatively translocated to the nucleus in response to VCR treatment (Fig. 8*E*). Attempts to verify these results using immunofluorescence microscopy were unsuccessful because the process of cytospinning the fragile primary cells onto slides resulted in morphological deformation that precluded accurate assessment of changes in the subcellular locations of AIF or EndoG.

### VCR induces autophagy which has a protective role

**Figure 9:**
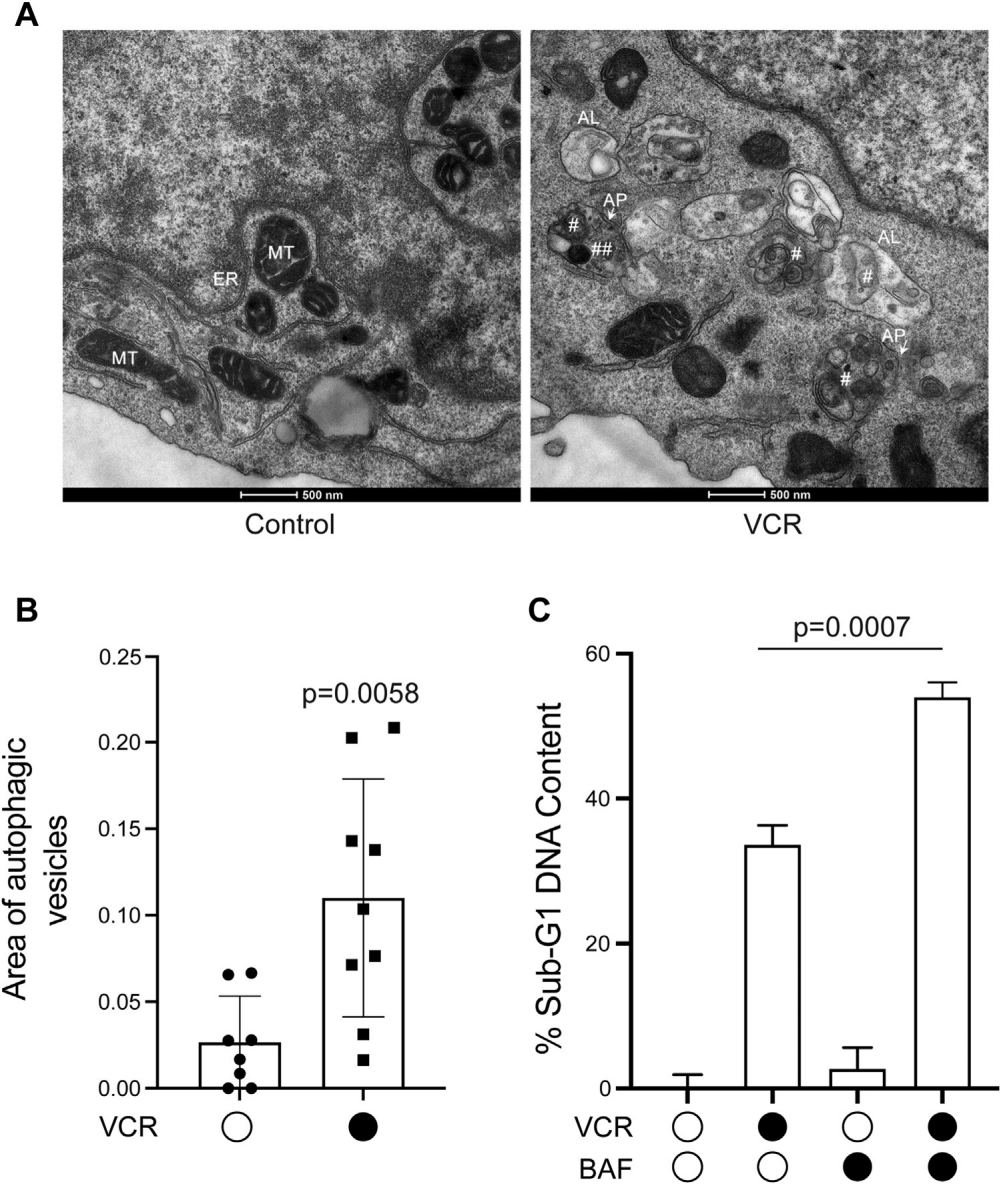
**Vincristine treatment of G1 phase ALL cells is associated with autophagy.***A*, representative electron micrographs of G1 phase ALL-5 cells treated with vehicle (0.1% DMSO) or 100 nM VCR for 12 h. MT, mitochondrion; ER, endoplasmic reticulum; AP, autophagosome; AL, autolysosome; #, undigested cytoplasmic content; ##, undigested cytoplasmic content with same electron density as mitochondrion. *B*, quantitation of autophagic vesicle area. Autophagic vesicle area, normalized to cytoplasmic area and expressed as a ratio, was determined for a total of eight images of control and nine images of VCR-treated G1 phase ALL-5 cells. Results are displayed as a scatter plot with mean and SD indicated. *C*, effect of the autophagy inhibitor bafilomycin A1 (BAF) on VCR-induced DNA fragmentation. G1 phase ALL-5 cells were treated with vehicle, 100 nM VCR, or 3 nM bafilomycin A1, alone and in combination, for 36 h, as indicated, and sub-G1 DNA content determined by propidium iodide staining and flow cytometry, as described in Experimental procedures. Results shown are mean ± SD, n = 3. ALL, acute lymphoblastic leukemia; VCR, vincristine.

We reasoned that further insight into the mechanism of G1 phase death in response to VCR might be obtained by closely examining intracellular morphology in vehicle and drug-treated cells. Given the problems associated with cytospinning, as indicated above, we elected to utilize electron microscopy, since processing of cells did not require centrifugation but instead entailed freezing and sectioning which preserved morphology. G1 phase ALL cells were prepared by centrifugal elutriation, treated with vehicle or VCR, and processed for visualization by electron microscopy, as described in Experimental procedures. Inspection of the images revealed that vehicle and VCR-treated cells differed strikingly with respect to the presence in the latter of double membraned structures, which were much less frequent in vehicle-treated cells. Representative images are shown in Figure 9*A*. The presence of autophagosomes (APs), defined as double membraned structures containing undigested cytoplasmic content which has not been fused with a lysosome (49), are readily evident after VCR treatment (Fig. 9*A*, right panel). Some of the material within APs had the same electron density as mitochondria (Fig. 9*A*, right), facilitating their identification. An increased number of autolysosomes (ALs) were also observed in VCR-treated cells (Fig. 9*A*, right). Quantitation of autophagic vesicle area normalized to cytoplasmic area, conducted as described in Experimental procedures, confirmed a significant increase after VCR treatment (Fig. 9*B*). These results suggested that VCR induced autophagy in G1 phase ALL cells. Control and VCR-treated G2/M cells were subjected to a similar analysis and an increased incidence of APs and ALs after VCR treatment was also observed (Fig. S5), indicating that autophagy may be a general response to microtubule depolymerization in this system.

Autophagy can function either to promote death or to promote survival (29). To determine whether autophagy played a role in cell fate in this context, bafilomycin A1 (50), which inhibits autophagy *via* targeting of the vacuolar-type H^+^-ATPase responsible for acidifying lysosomes, was tested. Because bafilomycin is cytotoxic, ALL cells were first treated with the compound alone to determine concentrations that were sublethal, and a concentration of 3 nM was selected. ALL cells in G1 phase were then prepared, treated with vehicle or VCR without or with cotreatment with bafilomycin, and after 36 h, sub-G1 DNA content determined. As shown in Figure 9*C*, cell death as determined by DNA fragmentation was highly significantly increased after cotreatment with VCR and bafilomycin *versus* treatment with VCR alone. Importantly, bafilomycin had very little effect when used alone, indicating that the enhancement observed with cotreatment was not simply due to additive effects on toxicity. These results indicate that autophagy functions to protect G1 phase ALL cells from cell death in response to microtubule depolymerization.

## Discussion

MTAs play a key role in the treatment of numerous types of cancer (1). Because mitotic spindle microtubules are highly dynamic (2), they are particularly sensitive to inhibition by MTAs, and as a consequence, most rapidly dividing cancer cell lines grown in culture are susceptible in M phase. However, in the clinical setting, many tumor types have protracted doubling times and low mitotic indices yet are still susceptible to MTAs (18, 19, 24). Thus, mitotic death may be less important *in vivo* than *in vitro*, and this raises important questions about how MTAs exert their cytotoxicity clinically, which in turn has intensified efforts to understand nonmitotic actions of MTAs (51). We have shown in detailed studies presented previously that primary ALL cells are susceptible to microtubule depolymerizing agents such as VCR not only in M phase but also specifically in G1 phase (25, 28). The ability to isolate unperturbed primary ALL cells highly enriched in either G1 or G2/M phases *via* centrifugal elutriation ((26) and Fig. 1) and, thus without the problems associated with drug-induced cell cycle synchronization, provided an ideal model system to compare and contrast the underlying death pathways.

Primary ALL cells in G2/M phases treated with VCR died in a manner characteristic of intrinsic apoptosis. Thus, we observed caspase-3 activation, Bax activation, loss of mitochondrial membrane potential, and nucleosomal fragmentation of DNA. However, DNA fragmentation in G2/M phase cells treated with VCR was not completely inhibited by caspase inhibition (Fig. 5), suggesting that death may result from the combined effects of intrinsic apoptosis and an additional caspase-independent mechanism. Death of G2/M phase ALL cells in response to VCR was also associated with phosphorylation of prosurvival Bcl-2 proteins. We have previously shown that in the context of mitotic death, cyclin-dependent kinase (Cdk) 1/cyclin B is largely responsible for Bcl-2 and Bcl-xL phosphorylation (30, 52), and plays a contributory role to Mcl-1 phosphorylation (33). A broader survey of cell lines indicated that these events represent a conserved mitotic death signature (53), with Cdk1/cyclin B-mediated phosphorylation inactivating the prosurvival functions of these Bcl-2 family members enabling apoptotic death. The results with G2/M phase cells (Fig. 2) strongly support our model that mitotic death is closely associated with phosphorylation of prosurvival Bcl-2 proteins. In contrast, VCR-induced death of ALL cells in G1 phase was not associated with phosphorylation of prosurvival Bcl-2 proteins (Fig. 2). Since Cdk1/cyclin B is not active in G1 phase (35), the lack of Bcl-2/Bcl-xL/Mcl-1 phosphorylation in the context of G1 phase death is consistent with expectations and further highlights a key difference in the two death pathways.

In contrast to G2/M cells, death of G1 phase cells in response to VCR lacked features of intrinsic apoptosis, and the low levels of caspase-3 and Bax activities observed were likely attributable to a minor proportion of cells that escaped and advanced to die in mitosis. Indeed, the characteristics of death of G1 phase cells are more similar to those described for parthanatos (41), with the occurrence of protein parylation and nuclear translocation of AIF, both of which were blocked by the PARP-1 inhibitor olaparib (Fig. 8). However, this conclusion is tempered by the lack of effect of olaparib on VCR-induced DNA fragmentation (Fig. 8*D*). A possible explanation lies in the well-documented redundancy of cell death pathways, where blocking one pathway results in cell death *via* another. For example, it has been shown that inhibition of caspases can cause a switch from apoptosis to necrosis or to autophagic death in specific contexts or conversely that inhibition of autophagy can promote caspase-dependent apoptosis (54). In VCR-treated G1 phase ALL cells, olaparib may block AIF-mediated DNA fragmentation, but other DNA cleavage mechanisms may operate, such as that mediated by EndoG, which also undergoes nuclear translocation (Fig. 8*E*). Further studies will be required to investigate the role of parylation and the relative contributions of AIF and EndoG, and perhaps other factors, to VCR-mediated DNA fragmentation in G1 phase cells. In addition, technical advancements and optimization are underway to improve detection of proteins in primary ALL cells by immunofluorescence to support these findings.

Although the majority of the studies reported here were performed using ALL-5 cells, ALL-2 cells showed a highly similar profile of caspase-3 activation after VCR treatment, with low levels in G1 phase cells and much higher levels in G2/M phase cells (Fig. S2). In addition, as shown in our previous study (25), ALL-5 and ALL-2 are susceptible to VCR in both G1 and M phases with similar levels of sensitivity and similar kinetics of cell death. While more detailed studies of other ALL cultures are needed, these observations strengthen the notion that the death pathways in response to VCR are conserved in independent ALL cultures.

PARP-1 activation typically occurs in response to DNA damage (41). This raises the question of how microtubule depolymerization might lead to DNA damage. It is recognized that interphase microtubules play an important role in trafficking proteins into the nucleus, including key regulatory proteins such as retinoblastoma protein (55) and p53 (56). In addition, it has been shown that MTAs enhance the toxicity of DNA damaging agents by blocking nuclear trafficking of proteins involved in DNA repair (57). In preliminary experiments using G1 phase ALL cells, we have similarly found that VCR disrupts nuclear import of several proteins including those which function in nucleotide excision repair†. A plausible sequence of events is that VCR, through microtubule depolymerization, prevents trafficking of DNA repair proteins to the nucleus of G1 phase ALL cells, which in turn generates DNA damage and activates PARP-1 and cell death *via* parthanatos. The fact that death of G1 phase ALL cells is associated both with protein parylation and with PARP cleavage is likely due to differing kinetics, with parylation prominent at a treatment time of 16 h (Fig. 8*B*) when PARP is still largely intact (Fig. 2).

Through electron microscopy, we made the interesting observation that VCR-treated G1 phase cells exhibited features of autophagy (Fig. 9). That bafilomycin augmented DNA fragmentation caused by VCR, yet had no effect alone, suggests that autophagy operates in this context to protect cells from the effects of VCR. As discussed above, death of G1 phase cells in response to microtubule depolymerization may result from defective nuclear accumulation of key proteins that are needed to prepare cells for S phase and to maintain DNA integrity. In the context of G1 phase death, autophagy may be initiated as a salvage pathway to protect cells from the initial adverse effects of VCR on protein homeostasis. Interestingly, evidence of autophagy was also observed in VCR-treated G2/M phase cells (Fig. S5). While the role in this context will require further exploration, other studies have shown that autophagy facilitates cell survival during mitotic arrest (58).

These observations have potential clinical implications. First, they suggest that autophagy inhibitors may augment the cytotoxicity of microtubule depolymerizing agents and encourage more detailed studies combining such agents. Second, a major obstacle in the use of MTAs clinically is the difficulty in stratifying patients, in order to determine which patients will benefit from MTA therapy *versus* patients who are unlikely to benefit but will nonetheless suffer often debilitating side-effects (59, 60). This requires evaluation of how individual tumors respond to therapy, which in turn depends on reliable biomarkers that report drug action at the tumor site. Current information suggests that for MTAs, interphase death is more clinically relevant than mitotic death (18, 19, 47), but whether the underlying mechanisms differed or were similar was largely unknown. The findings reported here indicate that the death pathway(s) activated in G1 phase *versus* M phase cells in response to microtubule depolymerization are fundamentally different. By providing insight into the specific pathways and proteins involved, the observations provide a basis to investigate and better define mechanisms that operate clinically and suggest potential biomarkers useful in reporting drug efficacy.

## Experimental procedures

### Materials

Iscove’s Modified Dulbecco’s Medium (SH30228) was obtained from HyClone Laboratories Inc. Cholesterol (C3045), amphotericin-B (A2942), iron(III) chloride hexahydrate (236489), and APO-transferrin (T1147) were from Sigma-Aldrich. Human serum albumin (HA1000) was from Golden West Biologicals. Glutamine (25-005) and penicillin/streptomycin (30-002) were obtained from Corning. Insulin (128-100) was obtained from Cell Applications. Phospho (Ser 62) Bcl-xL rabbit antibody (sc-101644), Mcl-1 mouse antibody (sc-12756), Bcl-2 mouse antibody (sc-509), active Bax mouse antibody (6A7) (sc-23959), cyclin B mouse antibody (sc-245), EndoG mouse antibody (sc-365359), protein A/G-agarose beads (sc-2003), and vincristine sulfate (sc-201434) were from Santa Cruz Biotechnology. Bax rabbit antibody (5023), Bcl-xL rabbit antibody (2762), MLKL rabbit antibody (14993), phospho-MLKL rabbit antibody (91689), GAPDH rabbit antibody (2118), and PARP rabbit antibody were from Cell Signaling Technology. AIF rabbit antibody (1998) and poly (ADP-ribose) polymer mouse antibody (14459) were from Abcam. JC-1 dye was purchased from Enzo Life Sciences, and FCCP was from Sigma Chemical Co. Caspase-3 activation assay (K106) was from BioVision Incorporated; Z-VAD-FMK (A1902), ABT-263 (A3007), and bafilomycin A1 (A8627) were from ApexBio; TNF-α (300-01A) was from PeproTech; cycloheximide (C104450) was from Sigma-Aldrich; and olaparib (A10111-25) was from Cedarlane Labs. HT-29 colon carcinoma cells (HTB38) were obtained from ATCC.

### Cell culture

Cultures of primary human B-ALL cells were used in this study. They were isolated from B-ALL patients and characterized as described previously (61). All experiments were performed using ALL-5 cell cultures, and confirmatory experiments assessing caspase-3 activation were performed using ALL-2 cell cultures (37). ALL-5 were originally designated PH cells, and ALL-2 were originally designated CM cells (61). ALL-5 and ALL-2 are B-ALL cells of the common immune phenotypic subtype and both display the t(9;22) chromosomal translocation. Cells were maintained in suspension in IMDM medium containing 10 μg/ml cholesterol, 6 mg/ml human serum albumin, 2 mM L-glutamine, 2% v/v amphotericin-B/penicillin/streptomycin, 1 μg/ml insulin, 200 μg/ml APO-transferrin, and 50 μM β-mercaptoethanol, as described previously (37), and passaged up to P30. KB-3 cells (HeLa subline), provided by Dr Michael Gottesman, and HT-29 cells were maintained in DMEM (5 g/L glucose), 1% penicillin/streptomycin/glutamine, and 10% FBS.

### Centrifugal elutriation

Centrifugal elutriation was performed using a Beckman JE-5.0 elutriation rotor as previously described (25, 26) with slight modifications. Briefly, ALL cells (4–6 ×10^8^) were suspended in 25 ml of elutriation buffer (Hank’s buffered salt solution containing 1.6 g/L 2-naphthol-6,8-disulfonic acid dipotassium salt and 2% FBS), passed through a 25G needle twice, and introduced into the elutriation chamber at a flow rate of 25 ml/min with a rotor speed of 3000 rpm. Rotor speed was reduced to 2920 rpm to collect the W1 wash fraction and then to 2620 rpm (661*g*) to collect the F1 fraction containing cells in G1 phase. Successive reductions in rotor speed were performed to collect wash fractions, and cells in G2/M phases were collected in the F3 fraction at 1860 rpm (333*g*). Aliquots of the fractions were subjected to propidium iodide staining to verify DNA content. Cells were resuspended in fresh growth medium.

### DNA content analysis

Cells were harvested and fixed in 70% ice-cold ethanol. DNA content was evaluated by propidium iodide staining according to the manufacturer’s instructions (BD Pharmigen), and flow cytometry was carried out by the UAMS Flow Cytometry Core Facility using a FACSCalibur (Becton Dickinson). The data were analyzed using the FlowJo (FlowJo, LLC) software.

### Cell extraction and immunoblot analysis

At least 10^7^ cells plated at a concentration of 1.25 × 10^6^ cells/ml were used for each condition. Whole cell extracts were prepared in the presence of protease and phosphatase inhibitors as previously described (25) and samples (25 μg protein per lane) analyzed by SDS-PAGE using 12% acrylamide gels followed by transfer to PVDF membrane. Membranes were probed with the following antibodies: phospho(Ser 62) Bcl-xL (1:1000), Mcl-1 (1:200), Bcl-xL (1:1000), PARP (1:2500), Bcl-2 (1:1000), Bax (1:2000), cyclin B (1:1000), phospho-MLKL (1:1000), MLKL (1:1000), PAR polymers (1:1000), AIF (1:2500), EndoG (1:1000), and GAPDH (1:10,000). Phospho(Ser62) Bcl-xL antibody was also used to detect mitotically phosphorylated Mcl-1 as described previously (50) and was readily distinguished on immunoblots by different molecular masses. Secondary antibodies against either mouse IgG (1:5000) or rabbit IgG (1:5000) were used dependent on the origin species of the primary antibodies. Apparent molecular weights derived from SDS-PAGE/immunoblotting of the proteins examined are as follows: Mcl-1, 40 kDa; Bcl-xL, 30 kDa; Bcl-2, 26 kDa; GAPDH, 37 kDa; PARP, 110 kDa; cleaved PARP, 85 kDa; Bax, 20 kDa; MLKL, 54 kDa; cyclin B, 55 kDa; AIF, 67 kDa; and EndoG, 32 kDa.

### Subcellular fractionation

All steps were conducted at 4 °C except as noted. Cells were washed in PBS and suspended in 0.4 ml lysis buffer (10 mM Hepes, pH 7.5, 10 mM KCl, 0.1 mM EDTA, plus protease, and phosphatase inhibitors) and after 15 min Triton X-100 was added to 0.5% followed by rapid mixing. Sample were then centrifuged at 12,000*g* for 10 min to obtain a nuclear pellet and an extranuclear supernatant. The nuclear pellet was suspended in 20 mM Hepes, pH 7.5, 0.4 M NaCl, 1 mM EDTA, plus protease and phosphatase inhibitors, incubated on ice for 30 min, and centrifuged at 16,000*g* for 10 min to obtain a nuclear extract.

### Bax activation assay

4 × 10^7^ cells plated at a concentration of 1.25 × 10^6^ cells/ml were used for each condition. Cells were pelleted from the culture medium, washed in PBS, and lysed at 4 °C in 1% CHAPS, 40 mM Hepes, pH 7.5, 120 mM NaCl, 1 mM EDTA, and 50 mM NaF, containing protease and phosphatase inhibitors, and protein quantified using the Bradford assay. Equivalent protein from each sample (450 μg) was incubated with 5 μg/mg protein active Bax antibody overnight at 4 °C. Samples were then incubated with protein A/G beads for 2 h at 4 °C, centrifuged, and washed three times with lysis buffer. After washing, beads were incubated with SDS sample buffer (containing 0.05 M DTT instead of β–mercaptoethanol) for 1 h at 37 °C. Immunoprecipitated samples were then centrifuged at 16,000*g* for 3 min and 20 μl analyzed by SDS-PAGE and immunoblotting for Bax. Whole cell extracts (20 μg) were analyzed similarly. Mock immunoprecipitation was performed by taking equal amounts (225 μg) of control and VCR-treated (48 h) extracts and subjecting the mixture to the same immunoprecipitation protocol but in the absence of active Bax antibody.

### Caspase-3 assay

At least 2 × 10^7^ cells plated at a concentration of 1.25 × 10^6^ cells/ml were used for each condition. After treatment, cells were harvested, and caspase-3 activity assessed using a Caspase-3/CPP32 Colorimetric Assay Kit from BioVision (K106). Briefly, cells were lysed, and protein quantified using the Bradford Assay. Protein (110 μg) from each sample was incubated with DEVD-pNA substrate for 2 h at 37 °C. After incubation, absorbance was read at 405 nM.

### DNA extraction and gel electrophoresis

To analyze small DNA fragments, 10^7^ cells were suspended in disruption buffer containing 10 mM Tris-HCl (pH 8.0), 100 mM NaCl, 1 mM EDTA 2% Triton X-100, and 1% SDS. After vigorous vortexing to ensure complete cell lysis, the lysate was extracted sequentially with phenol, phenol–chloroform, and lastly chloroform. Nucleic acids were ethanol precipitated and resuspended in Tris-EDTA containing 100 μg/ml RNAse A and incubated at 37 °C for 30 min. The resulting DNA was quantified using a Nanodrop 2000c spectrophotometer (ThermoScientific). DNA samples (8 μg) were separated on 2% agarose gels in Tris acetate–EDTA buffer. GeneRuler 100 bp DNA ladder (SM0243, ThermoFisher Scientific) was used to provide molecular mass standards. To analyze larger DNA fragments, 10^7^ cells were suspended in Proteinase K digestion buffer containing 50 mM Tris-HCl (pH 8.0), 100 mM NaCl, 5 mM EDTA, 2 mM CaCl_2_, 1% SDS, and 200 μg/ml Proteinase K. Cell lysates were digested at 55 °C overnight. DNA samples (2 μg) were separated by pulsed field gel electrophoresis using a 1.2% agarose gel containing pulsed field certified agarose in 0.5 × TBE (Tris-borate-EDTA) buffer, with an initial switch time of 1.5 s and a final switch time of 3.5 s for 12 h at 6 V/cm. Lambda HindIII (N3012S, New England Biolabs) was used to provide molecular mass standards. DNA was visualized by staining gels with 0.5 μg/ml ethidium bromide.

### Measurement of mitochondrial membrane potential

Loss of mitochondrial membrane potential was determined by monitoring changes in fluorescence of the cationic JC-1 dye. FCCP (10 μM), a protonophore which depolarizes mitochondrial membranes, was used as a positive control. After drug treatment, phase-separated cells (2 × 10^5^ per well) were washed in PBS and resuspended in Newmeyer buffer (0.3 M trehalose, 10 mM Hepes-KOH pH 7.7, 80 mM KCl, 1 mM EGTA, 1 mM EDTA, 0.1% BSA, and 5 mM succinate). Dye mastermix was prepared (4 μM JC-1, 0.2% digitonin, 40 μg/μl oligomycin, 20 mM β-mercaptoethanol in Newmeyer buffer) and allowed to incubate in the dark for 10 min. After incubation, an equal volume of dye mastermix was added to cell suspensions and incubated in the dark for 10 min. Aliquots of 30 μl were added to the wells of a black, clear bottom 96-well plate containing 30 μl of Newmeyer buffer. Fluorescence was measured at an excitation wavelength of 530 nm and an emission wavelength of 590 nm every 5 min for 2 h. Each condition was conducted in triplicate.

### Electron microscopy

ALL-5 cells in G1 phase or G2/M phases were obtained by centrifugal elutriation as described above and treated with vehicle or 100 nM VCR for 12 h or 6 h, respectively. After washing in PBS, cells were fixed in 2.5% glutaraldehyde/0.05% malachite green in 0.1 M sodium cacodylate buffer. Cells were poststained successively with 1% osmium tetroxide/0.8% potassium hexacyanoferrate (III) in 0.1 M sodium cacodylate buffer, then 1% tannic acid in molecular grade water, and then 0.5% uranyl acetate in molecular grade water before being dehydrated by incubation in a graded ethanol series followed by 100% propylene oxide. Embedding was performed in Araldite-Embed 812 (Electron Microscopy Sciences). 50 nm sections were collected on copper mesh grids using a Leica UC7 microtome and poststained with 0.5% uranyl acetate in 0.05 M sodium maleate buffer and then with Reynolds lead citrate. Images were collected using a Tecnai F20 (FEI) transmission electron microscope at 80 kv. For quantitation of autophagic vesicles, images from control cells and VCR-treated cells were examined for their presence which were recognized by double-membrane structures containing undigested cytoplasmic content. The area taken up by autophagic vesicles was determined and expressed as a ratio *versus* cytoplasmic area for each image and expressed as a scatter plot. ALs were also observed and recognized by a single limiting membrane surrounding cytoplasmic material undergoing degradation.

### Statistical analysis

Data were analyzed by Student’s *t* test with *p* < 0.05 being considered significantly different. All experiments were conducted with replicates of at least three and data reported as mean ± SD. All experiments have been repeated at least twice with essentially identical data.

## Data availability

All data are contained within the manuscript.

## Supporting information

This article contains supporting information.

## Conflict of interest

The authors declare they have no conflicts of interest with the contents of this article.

## Footnotes

† M. Delgado and T. C. Chambers, unpublished observations.
