# Supplementary material for: Primary acute lymphoblastic leukemia cells are susceptible to microtubule depolymerization in G1 and M phases through distinct cell death pathways
Source: J Biol Chem. 2022 Apr 15;298(6):101939. doi: 10.1016/j.jbc.2022.101939 (PMC9123221; doi:10.1016/j.jbc.2022.101939)
Supplement: Supporting Figures S1–S5 [file mmc1.docx]

Supporting Material

for

Primary acute lymphoblastic leukemia cells are susceptible to microtubule depolymerization in G1 and M phase through distinct cell death pathways

Magdalena Delgado, Randall R. Rainwater, Billie Heflin, Alicja Urbaniak, Kaitlynn Butler, Mari Davidson, Reine M. Protacio, Giulia Baldini, Andrea Edwards, Megan R. Reed, Kevin D. Raney,

and Timothy C. Chambers


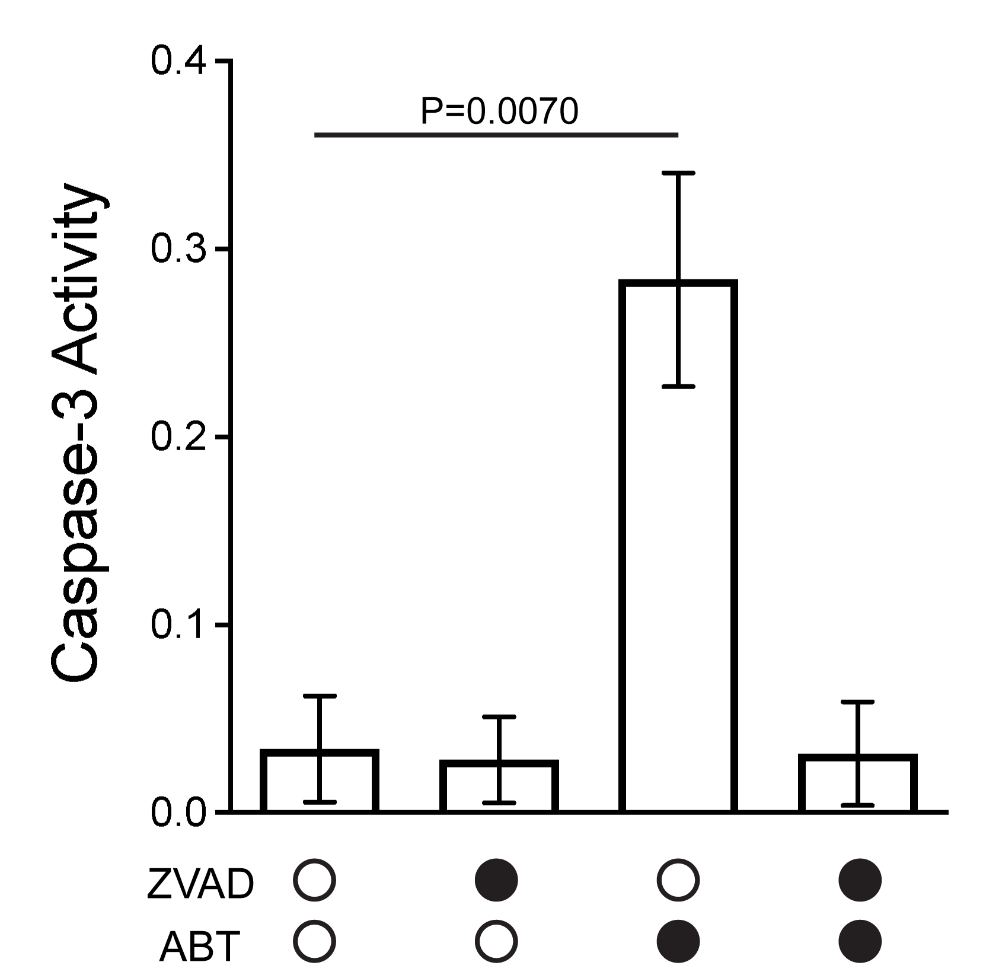


**Figure S1. Caspase-3 activation by ABT-263**. Asynchronous ALL-5 cells were treated with 100 nM ABT-263 (ABT) alone or in combination with 100 µM of Z-VAD-FMK (ZVAD) for 6 h, as indicated. Protein extracts were prepared and caspase-3 activity determined as described in Experimental Procedures. Data shown are mean ± S.D. (n ≥ 3) with the relevant p value indicated.

**
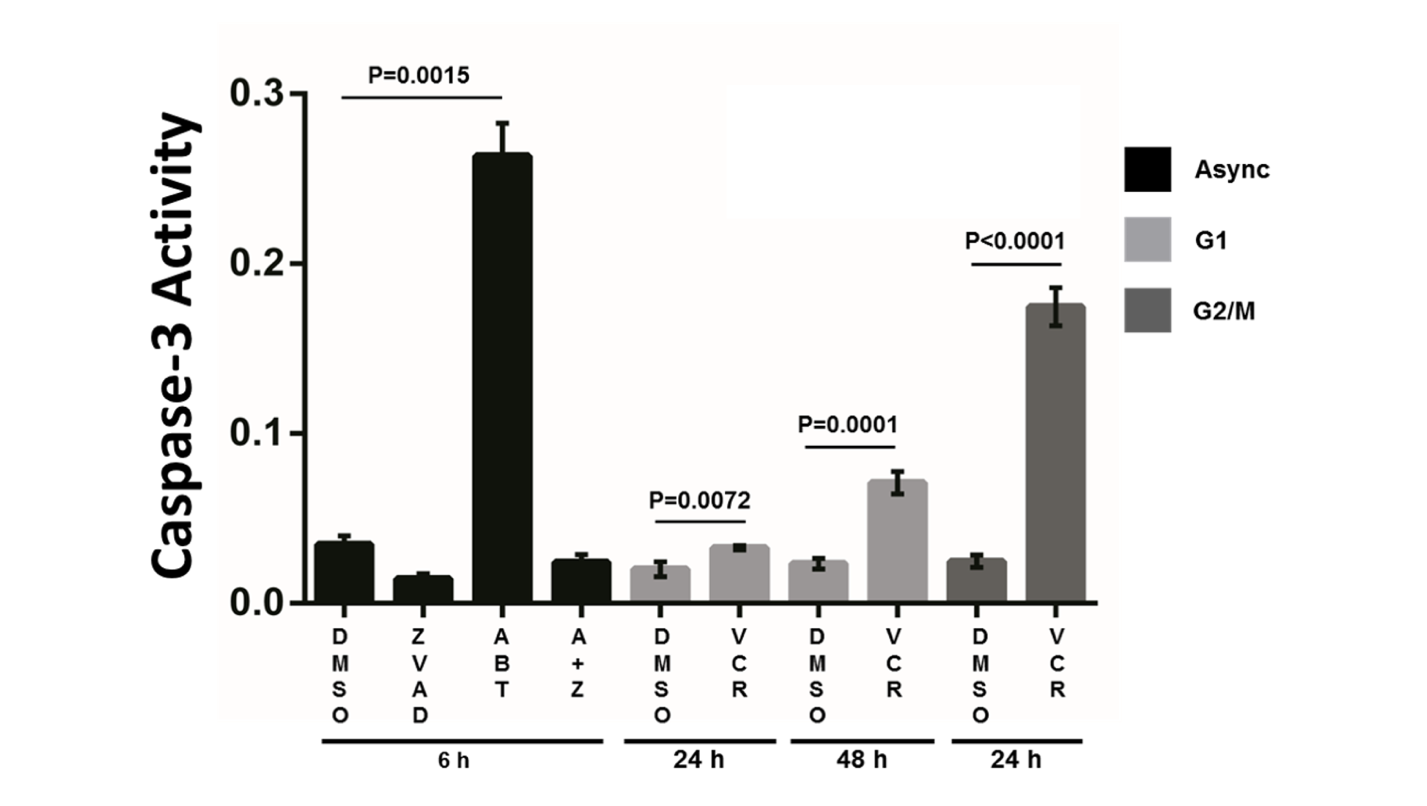
**

**Figure S2. Vincristine activates caspase-3 more strongly in G2/M than G1 phase ALL-2 cells.** Asynchronous (Async) ALL-2 cells were treated with vehicle (0.1% DMSO) or 100 nM ABT-263 (ABT, A) alone or in combination with 100 µM of Z-VAD-FMK (ZVAD, Z) for 6 h. G1 phase and G2/M phase ALL-2 cells were isolated by centrifugal elutriation and treated with 0.1% DMSO or 100 nM vincristine (VCR) for the times indicated. Protein extracts were prepared and subjected to caspase-3 assay as described in Experimental Procedures. Data shown are mean ± S.D. (n ≥ 3) with p values indicated.


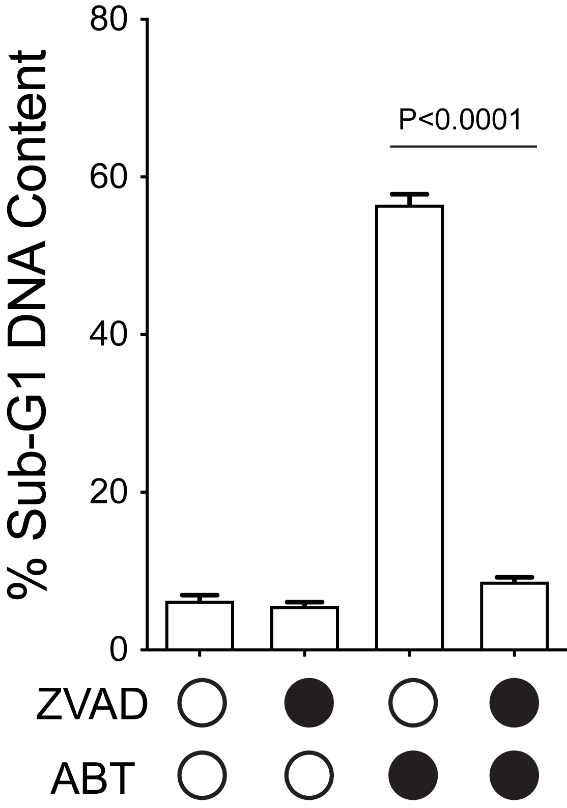


**Figure S3.** **Effect of caspase inhibition on DNA fragmentation induced by ABT-263.** Asynchronous ALL-5 cells were treated with 100 nM ABT-263 (ABT), 100 µM Z-VAD-FMK (ZVAD), or vehicle (0.1% DMSO), alone or in combination as indicated, for 6 h. Cells were harvested and stained with propidium iodide and analyzed for DNA content as described in Experimental Procedures. Data shown represent percent of cells with sub-G1 DNA content (mean ± S.D., n = 3) with the relevant p value indicated.

**
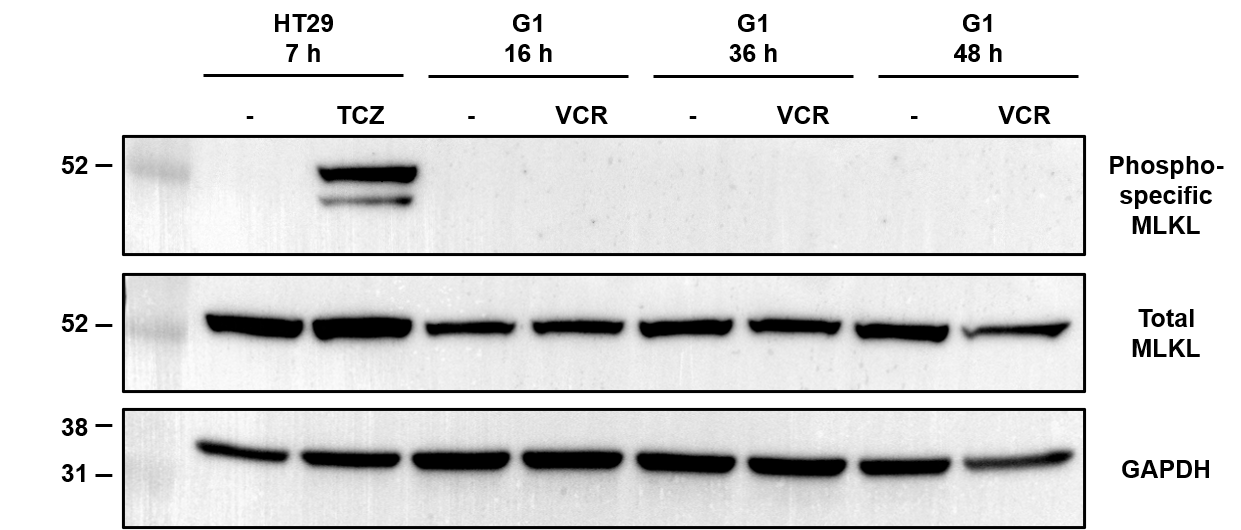
**

**Figure S4. Vincristine treatment of primary ALL cells in G1 phase is not associated with induction of necroptosis.** Whole cell extracts were prepared from HT-29 cells treated with vehicle (0.1% DMSO) or TCZ (TNF-α, 25 ng/mL; cycloheximide, 5 µg/mL; and Z-VAD-FMK, 20 µM) or from G1 phase ALL-5 cells treated with 0.1% DMSO or 100 nM vincristine (VCR) for the times indicated. Immunoblot analyses were performed for phospho-specific MLKL, total MLKL, or GAPDH as a loading control. Molecular mass standards (in kDa) are shown on the left.


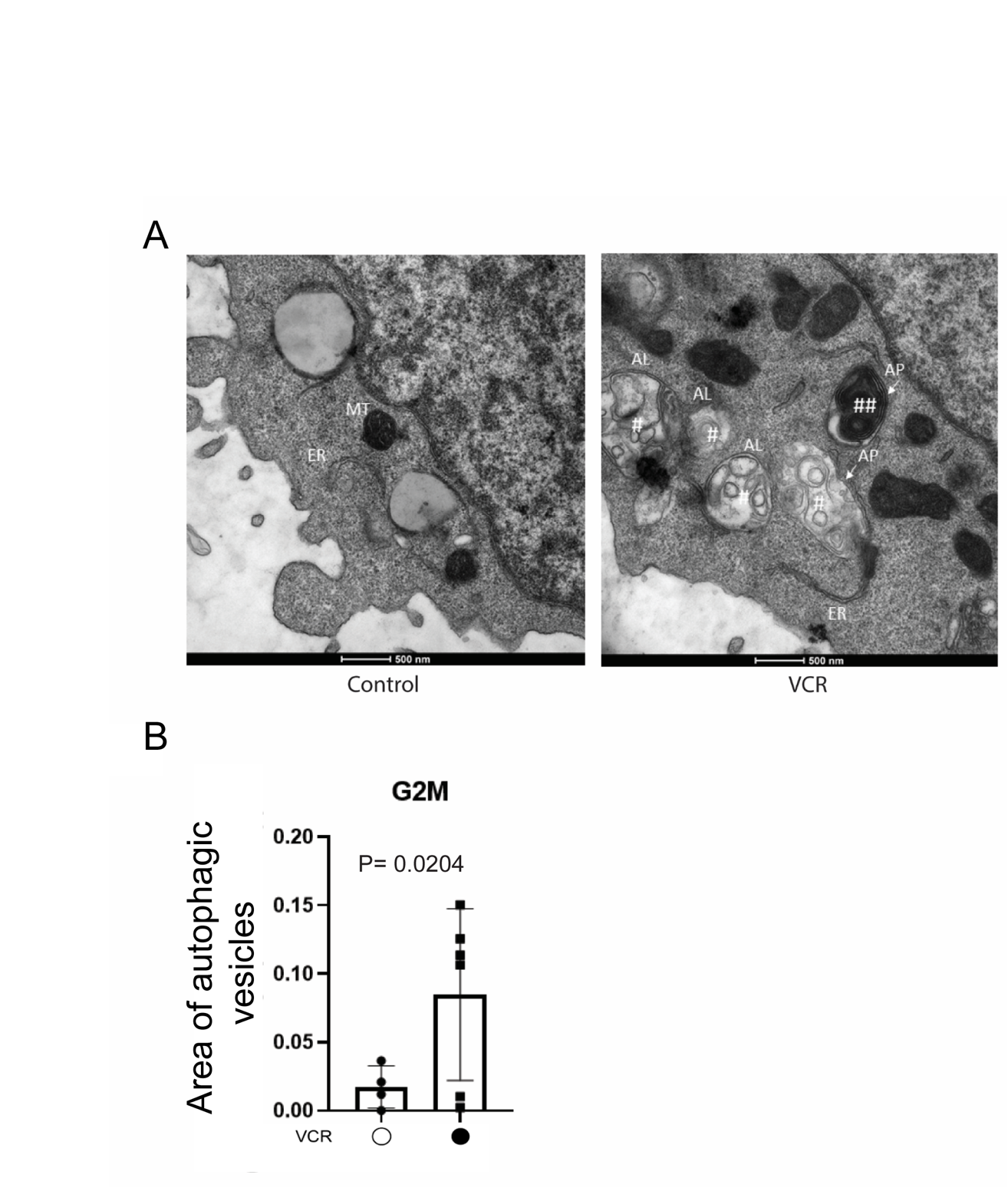


**Figure S5.** **Vincristine induces autophagy in G2/M phase ALL-5 cells.** A, representative electron micrographs of G2/M phase ALL-5 cells treated with vehicle (0.1% DMSO) or 100 nM VCR for 6 h. MT, mitochondrion; ER, endoplasmic reticulum; AP, autophagosome; AL, autolysosome; #, undigested cytoplasmic content; ##, undigested cytoplasmic content with same electron density as mitochondrion. B, Quantitation of autophagic vesicle area. Autophagic vesicle area, normalized to cytoplasmic area with values presented as a ratio, was determined for a total of 4 images of control and 6 images of VCR-treated G2/M phase ALL-5 cells. Results are displayed as a scatter plot with mean and S.D. indicated.
